# Continuous Drug Release by Sea Anemone *Nematostella vectensis* Stinging Microcapsules

**DOI:** 10.3390/md12020734

**Published:** 2014-01-27

**Authors:** Yossi Tal, Ari Ayalon, Agnesa Sharaev, Zoya Kazir, Vera Brekhman, Tamar Lotan

**Affiliations:** 1 StarletDerma Ltd., 8 HaEshel St., Caesarea 38900, Israel; E-Mails: Yossi@Starletderma.com (Y.T.); Ari@Starletderma.com (A.A.); agnesa@starletderma.com (A.S.); zoya@starletderma.com (Z.K.); 2 Marine Biology Department, The Leon H.Charney School of Marine Sciences, University of Haifa, Mount Carmel, Haifa 31905, Israel; E-Mail: vbrekhman@univ.haifa.ac.il

**Keywords:** cnidocyst, *Nematostella vectensis*, transdermal, diffusion cell, hydrophilic drug

## Abstract

Transdermal delivery is an attractive option for drug delivery. Nevertheless, the skin is a tough barrier and only a limited number of drugs can be delivered through it. The most difficult to deliver are hydrophilic drugs. The stinging mechanism of the cnidarians is a sophisticated injection system consisting of microcapsular nematocysts, which utilize built-in high osmotic pressures to inject a submicron tubule that penetrates and delivers their contents to the prey. Here we show, for the first time, that the nematocysts of the starlet sea anemone *Nematostella vectensis* can be isolated and incorporated into a topical formulation for continuous drug delivery. We demonstrate quantitative delivery of nicotinamide and lidocaine hydrochloride as a function of microcapsular dose or drug exposure. We also show how the released submicron tubules can be exploited as a skin penetration enhancer prior to and independently of drug application. The microcapsules are non-irritant and may offer an attractive alternative for hydrophilic transdermal drug delivery.

## 1. Introduction

The phylum Cnidaria, comprising sea anemones, corals, jellyfish and hydra, is one of the most ancient extant multicellular phyla, dating back about 700 million years [1]. It is characterized by stinging cells containing of nematocysts, microcapsules equipped with an injection system [2]. The nematocyst incorporates a tightly folded, inverted thin tubule, which upon activation is released at an ultrafast acceleration of 5 × 10^6^ *g* [3]. The discharge is controlled by changes in osmotic balance. Large aggregates of poly-γ-glutamate and metal cations are trapped within the microcapsules and serve as the internal “battery” of the system [4,5]. Water flowing through the porous net wall of the activated microcapsule causes the aggregated poly-γ-glutamate to dissociate. This in turn increases the osmotic pressure to 150 bars, resulting in ejection of the tubule and continuous injection of the microcapsular contents until the poly-γ-glutamate is fully swept out of the microcapsule [4]. About 30 subtypes of cnidocysts are known; though differing in size and shape, all were suggested to function according to the same basic principle [6]. We have previously demonstrated that the ultra-fast characteristic of the nematocysts can be leveraged to develop a natural microinjection system for immediate transdermal drug delivery with the microcapsular shell serving as the syringe barrel, the tubule as the syringe needle, and poly-γ-glutamate aggregate as the plunger. The system was tested using microcapsules isolated from the sea anemone *Aiptasia diaphana* [7,8,9].

Transdermal delivery has many advantages over conventional drug delivery systems. It reduces first-pass metabolism, eliminates drastic drug fluctuations, prevents adverse side effects related to gastric pH, decreases intravenous risks and increases patient compliance. However, only a relatively small number of drugs can be introduced transdermally [10,11] as the outermost layer of the skin, the stratum corneum, acts as a barrier, permitting penetration and delivery of small lipophilic drugs but not of compounds that are large or hydrophilic [10,12,13]. The cnidocyst system overcomes this limitation, thereby integrating the benefits of of rapid permeation of the skin barrier with high patient compliance. Furthermore, the system is specifically designed to deliver hydrophilic compounds [9].

Recently, the starlet sea anemone *Nematostella vectensis* emerged as a cnidarian model [14,15] and its genomic sequence was published [16], providing the necessary tools for molecular analysis [17,18]. In other studies, the types of *N. vectensis* nematocysts were characterized [19,20] and their protein content analyzed [21]. Cultivation of *N. vectensis* is straightforward, and its sexual reproduction can be controlled in the laboratory. It is therefore relatively easy to maintain large cultures, making this species an attractive source of nematocysts for drug delivery [22,23].

The aim of this work was to investigate the potential use of the *N. vectensis* nematocyst system as a vehicle for continuous transdermal drug delivery. Specifically, we examined (i) active skin penetration and continuous nicotinamide delivery; (ii) use of the nematocyst system as a skin penetration enhancer by differentiating between the activation and the drug application steps; and (iii) potential skin irritation by the nematocyst system.

## 2. Results and Discussion

### 2.1. Preparation of Nematocysts for Skin Penetration

**Figure 1:**
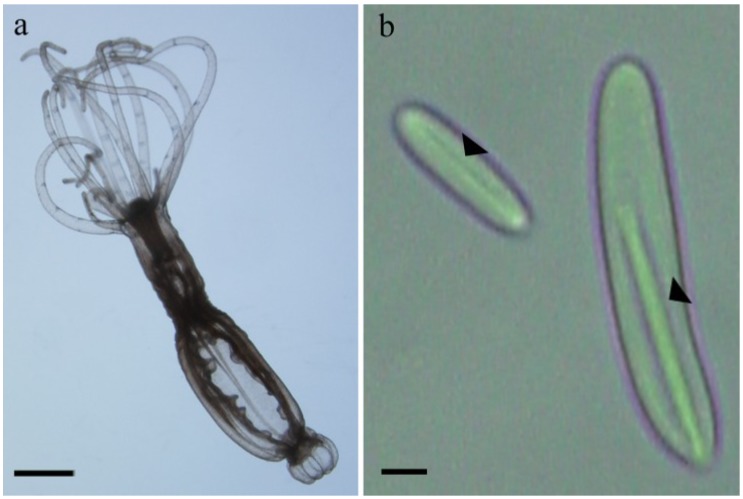
(**a**) The starlet sea anemone *N. vectensis*; scale bar 2 mm; (**b**) Two types of *N. vectensis* nematocysts. Arrow points to the packed tubule within the microcapsule; scale bar 2 µm.

*N. vectensis* contains three different types of microcapsules (basitrichous haplonema, microbasic mastigophores and spirocyst) [19]. However, during the microcapsules isolation process only two penetrant types of the microcapsules remained intact (basitrichous haplonema and microbasic mastigophores). The third type (spirocyst), which is more related to adhesion than penetration, discharge and is removed from the preparation with the cell debris. Repeated counts of the microcapsules distribution at the final preparation reveal a mixture of the two types of intact microcapsules containing approximately 35% of small cylindrical microcapsules (basitrichous haplonema) about 12 µm long and 2 µm in diameter and 65% of larger cylindrical microcapsules (microbasic mastigophores) about 20 µm long and 3 µm in diameter (Figure 1). We have previously demonstrated that added cations are needed for microcapsular stability and activation [8]. Therefore, the isolated preparation was freeze-dried in the presence of NaCl and CaCl_2_. The latter compound was needed to prevent the microcapsules from discharging spontaneously, despite our earlier finding that divalent ions can interfere with the activation. The preparation was kept dry, and was formulated into an anhydrous gel before being applied over the skin. The microcapsular preparation was designed to be activated with water, but unlike in our previous experiments with *A. diaphana* microcapsules [8,9] only 60% activation was achieved here. This suggested that Ca^++^ ions, which have 50- to 100-fold higher affinity for the pγGlu matrix than monovalent Na^+^ [4,24], interfere with the discharge process. As we could not prepare active microcapsules without Ca^++^ we tested activation with several chelators (data not shown). Pentasodium triphosphate was identified as the most active chelator, increasing activation up to 95% within 10 min. Thereafter all experiments were carried out with pentasodium triphosphate as activator.

**Figure 2:**
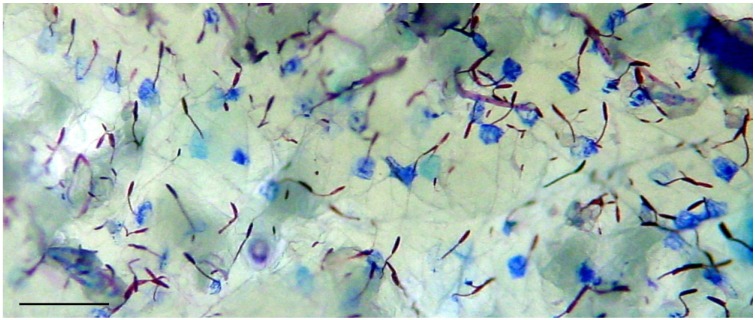
Delivery of dye compound to the skin; Gel containing microcapsules was spread over isolated nude mouse skin and activated in the presence of 0.05% toluidine blue dye solution for 10 min. After rinsing of the skin with DDW, blue spots in the skin are clearly seen; scale bar, 100 µm.

To examine whether the purified microcapsule powder (see Experimental Section) had retained its potential ability to penetrate hard substances such as the skin and also deliver an exogenous compound transcutaneously, we applied the microcapsular gel over nude mouse skin *in vitro* and activated it in the presence of toluidine blue dye. After 10 min, the skin was rinsed with DDW and photographed. The microcapsules discharged and their tubules pierced the skin, leaving a blue spot at their penetration point (Figure 2). Washing did not remove this coloration, which remained mainly at the tubule penetration point, indicative of transcutaneous delivery. A closer look revealed that only small part of the tubule length, up to 40 µm out of tubule lengths of 100 and 180 µm, had penetrated the skin. This depth, together with a penetration point of less than 0.5 µm, is a virtually noninvasive application. We concluded that an isolated microcapsular preparation of *N. vectensis* in a hydrophilic solution containing a Ca^++^ chelator as an activator is sufficient to mimic the natural discharge characteristics of these microcapsules.

### 2.2. Nicotinamide Permeation across Nude Mouse Skin *in Vitro*

To quantitate the delivery parameters of the *N. vectensis* microcapsule preparation we used the Franz diffusion cell system for measuring the amount of nicotinamide delivered *in vitro* through the full-thickness skin of a nude mouse. We chose this preparation as it is a convenient and readily available model for percutaneous penetration [25], and has been found to serve as a suitable skin model for testing nematocysts [8]. Nicotinamide, a water-soluble amide of nicotinic acid (niacin), acts as an antioxidant and an anti-inflammatory agent, and is a potent immunomodulator. In clinical practice it is used to treat variety of inflammatory skin disorders including bullous pemphigoid, acne, and rosacea, and it also helps to protect the skin against reactive oxygen species induced by UV irradiation [26,27]. We used nicotinamide as a model drug to investigate, first, whether our preparation of *N. vectensis* microcapsules can actually deliver a drug subcutaneously and what concentration of microcapsules is needed; and secondly to examine whether the released tubules, once opened, can serve as channels for continuous release, so that longer exposure to the drug would then result in its higher subcutaneous concentration.

**Figure 3:**
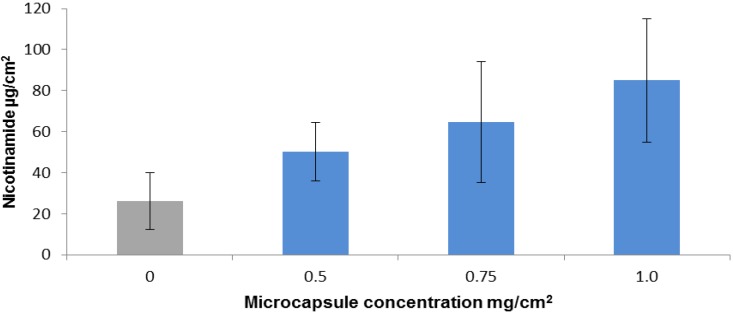
Accumulation of actively delivered nicotinamide as a function of microcapsule concentration. 4% Nicotinamide in activator solution was added to gel formulations containing 0 (control), 0.5, 0.75 or 1 mg microcapsules per square centimeter of skin (*n* ≥ 6). After 1 h the drug was removed and the skins were left in the diffusion cell for up to 5 h to allow subcutaneous diffusion of the delivered nicotinamide. Error bars represent means ± SD. The amount of nicotinamide delivered by the microcapsule gel was significantly greater than the amount delivered by the control (*p* < 0.001).

**Figure 4:**
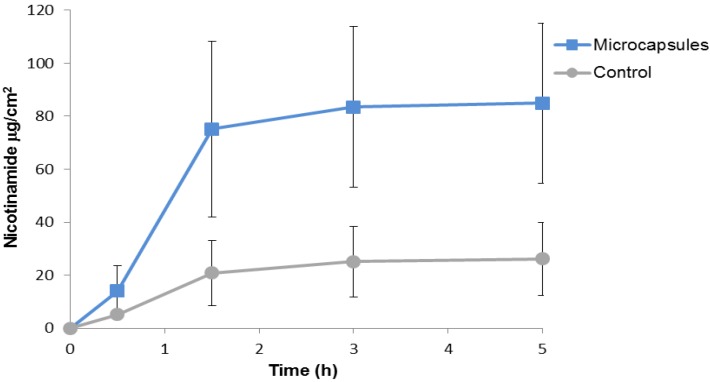
Kinetics of cumulative nicotinamide under the skin. Microcapsule gel (1 mg capsules per square centimeter of skin; 1 mg/cm^2^ skin) (*n* ≥ 6) or control without microcapsules (*n* = 8) was spread over the skin and nicotinamide (4% in activation solution) was added. After 1 h the nicotinamide was removed and the skins were left in the diffusion cell for up to 5 h. Samples were taken after 0.5, 1.5, 3 and 5 h. Error bars represent means ± SD. The amount of nicotinamide delivered by the microcapsules was approximately fourfold greater than in the control (*p* < 0.001).

For the first investigation, *i.e.*, to find out if nicotinamide, when added to activator solution (1% pentasodium triphosphate), can be delivered under the skin, we applied the mixed drug-activator solution with microcapsule gels at three different concentrations and with control gel without microcapsules. Nicotinamide was left for 1 h, after which the application was washed off the upper skin surface to remove any traces of the drug. The skin pieces were then left in the diffusion cells for an additional 4 h to allow the delivered nicotinamide to diffuse from the skin to the lower reservoir under the skin for analytical analysis. We found that the amount of nicotinamide delivered transcutaneously was increased as a function of microcapsular concentration (Figure 3). The amount of nicotinamide delivered at the lowest concentration of microcapsules was twice the amount delivered in the control gel, and at the highest microcapsular concentration it was 4-fold higher than in the control. Analysis of the kinetics of percutaneous penetration by the microcapsules showed that after 90 min about 90% of the delivered drug had already reached the compartment under the skin (Figure 4).

**Figure 5:**
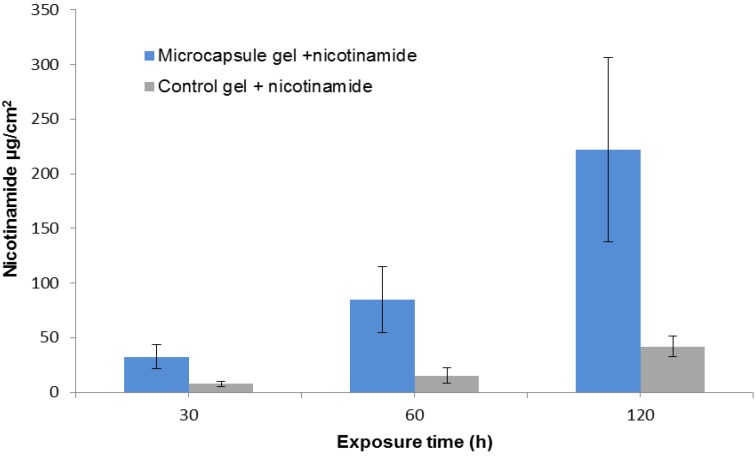
Cumulative permeation of nicotinamide as a function of its exposure duration. Nicotinamide (4% in activator solution) was added to microcapsule gel (1 mg/cm^2^ skin) or to control gel without microcapsules for 30, 60, or 90 min (*n* ≥ 6). The drug was then removed and the skins were left in the diffusion cell for up to 5 h. Error bars represent means ± SD. The amount of delivered nicotinamide was significantly greater in the skin treated with microcapsule gel than in the control (*p* < 0.001).

To test our hypothesis that the released tubules can function as a skin penetration enhancer we examined the effect of drug exposure time on drug accumulation. Using a constant microcapsular concentration of 1 mg/cm^2^ skin, we applied nicotinamide for 30, 60, or 90 min, then washed the skins as in the preview experiments and left them in the diffusion cell for up to 5 h. The amount of nicotinamide delivered by the microcapsule gel was increased by about seven fold between the exposure periods of 30 and 120 min, and was 4 to 5.5 times greater than control (Figure 5).

These experiments demonstrated that *N. vectensis* microcapsules can actively and transcutaneously deliver the drug in activation solution. The results also implied that the ejected tubules remain open, allowing continuous diffusion of the hydrophilic drug through the open channels, thus reaching a much higher concentration than in the control.

### 2.3. *In-Vitro* Delivery of Lidocaine Hydrochloride Using Applied Microcapsules as a Penetration Enhancer

**Figure 6:**
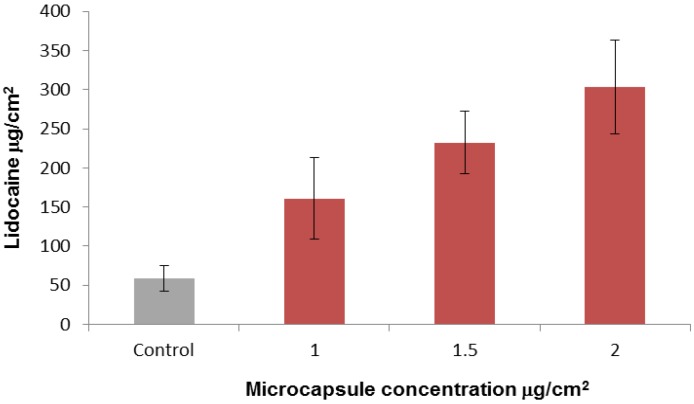
Cumulative permeation of lidocaine delivered passively by discharged microcapsules. Lidocaine HCl 5% was added for 1 h after activation (10 min) of gel formulation containing microcapsules at 0 (control) 0.5, 0.75 and 1 mg/cm^2^ skin (*n* ≥ 6). After 1 h the lidocaine HCl was removed, and the skins were left in the diffusion cell for up to 5 h to allow subcutaneous diffusion of the delivered lidocaine. Error bars represent means ± SD. The amount of lidocaine delivered by the microcapsule gel was significantly greater than the control (*p* < 0.001).

The successful results of the drug application described above necessitated compatibility of the drug with the basic pH of the activation solution. To overcome this potential limitation of this method of drug delivery, we separated the first step (microcapsule activation) and the second step (drug application). Lidocaine hydrochloride was chosen as the model drug for this experiment. Lidocaine is mostly used in its nonhydrophilic phase at a basic pH as a topical formulation for local anesthesia, whereas we tested it here in its hydrophilic acidic phase as lidocaine hydrochloride (lidocaine HCl). As before, we tested application of the drug in the diffusion cell system using full mouse skin. Microcapsule gels at three concentrations and control gel without microcapsules were applied to the skins and activated for 10 min. Thereafter, the activation solution was removed and 5% lidocaine HCl was applied. After 1 h the drug was washed out and the skins were left in the diffusion cell for an additional 4 h. The results showed that lidocaine was delivered passively through the microcapsule system and that the amount of drug delivered was proportional to the microcapsule concentration (Figure 6). Furthermore, depending on the microcapsule concentration about 2.5-fold to 5-fold more lidocaine accumulated in the microcapsule gel applications than in the control.

The above results show that the microcapsules gel formulation can serve as a drug penetration enhancer before application of a hydrophilic drug. Skin penetration enhancers can be divided functionally into two groups: one that targets the drug by means of an advanced formulation and the other consisting of chemical or physical enhancers that modulate the skin. The first group, which is drug specific, usually exploits lipophilic permeants and is not compatible with hydrophilic drugs [28,29]. In the second group, lipids and/or proteins of the stratum corneum are modified by solvents, surfactants or other formulations, which usually are ineffective for delivery of hydrophilic compounds [29]. The physical enhancers are more compatible with hydrophilic drugs than the chemical enhancers but they usually require the use of devices such as iontophoresis, ultrasound, mechanical abrasion, or microneedles. As a physical enhancer, application by the microcapsules appears to resemble physical enhancement by hollow microneedles [30]. However, a comparison between them shows that microcapsules have a number of advantages: their submicron diameter, compared to the microneedles diameter of 25–200 μm, their noninvasive penetration depth of 40 μm compared to 0.1–1 mm for microneedles and the method of application by topical formulation, compared to the need for a device to achieve uniform pressure for insertion of the microneedles tip into the skin [12,31].

### 2.4. *In-Vitro* Skin Irritation Test

To evaluate the safety of the microcapsule preparation we performed a skin irritation test *in vitro* using human epidermis reconstructed with EpiDerm tissues. After exposing the tissues for 1 h to microcapsule gel (1 mg/cm^2^ skin) or to a control gel without microcapsules, we carried out a cell viability test. The tested preparation and the control were found to be similarly non-irritant (see Experimental Section).

## 3. Experimental Section

### 3.1. Isolation of Microcapsules

Intact microcapsules were isolated as previously described, with minor modifications [19,21]. Briefly, *N. vectensis* were collected and kept frozen before microcapsule extraction. The anemones were homogenized in 12.5 ppt artificial seawater (their medium of cultivation), and this was followed by two centrifugations in Percoll gradients differentiating between the relatively dense intact microcapsules and the discharged microcapsules and the cell debris. The isolated purified microcapsules were washed with decreasing salinity of NaCl and CaCl_2_ to a final concentration of 15 mM NaCl and 0.2 mM CaCl_2_ and immediately freeze-dried. The microcapsules were kept in powder form at 2 to 8 °C until use.

### 3.2. Chemicals and Formulations

Microcapsule powder was mixed with anhydrous topical gel of 2% hydroxypropylcellulose in absolute ethanol as previously described [8,9]. We applied different quantities of microcapsules in a constant amount of gel (8 mg), ranging from 0.5 to 2.0 mg dry weight of microcapsules per square centimeter of skin (mg/cm^2^). Nicotinamide (pyridine-3-carboxamide) at 4% was used in an activating solution of 1% pentasodium triphosphate (final pH, 8.5). For microcapsule activation without the drug (control) we used 1% pentasodium triphosphate pH 9.3. Lidocaine HCl (2-(diethylamino)-*N*-(2,6-dimethylphenyl) acetamide hydrochloride) was used at 5%, pH 6.3. The lidocaine-HCl solution was prepared every week and kept at 2 to 8 °C until use.

### 3.3. Diffusion Cell Method

Permeability of the tested compounds through the full skin of a nude mouse was measured *in vitro* in a Franz diffusion cell system (PermeGear V3, PermeGear, Hellertown, PA, USA) at 35‒37 °C as previously described [8], with the following modifications. Microcapsule powder mixed with 8 mg of gel was introduced into the donor chambers on the skin and overlaid with either nicotinamide formulation or activator pentasodium triphosphate solution. As a control, skin pieces were covered with gel without microcapsules and were overlaid with the same drug/activator solution. The nicotinamide formulation was left for 30 min to 2 h and the drug was then thoroughly washed out. Alternatively, for skin penetration enhancement the activator was applied for 10 min and then replaced by lidocaine HCL, which was left on the skin for 1 h. The skins were then left in the diffusion cell for up to 5 h to allow diffusion of the delivered drug under the skin. During that period, samples were taken from the receiver compartment after 0.5, 1.5, 3 and 5 h and analyzed by high-performance liquid chromatography (HPLC).

### 3.4. HPLC Analysis

Lidocaine and nicotinamide contents were analyzed in a Hewlett-Packard HP-1090 HPLC system equipped with an autosampler and a diode-array UV detector. Lidocaine analysis was performed as previously described [8]. Nicotinamide was analyzed using a Kromasil 100-5-C18 column (250 × 4.6 mm), mounted on a guard column (Thermo Hypersil Gold Drop-in guars 10 × 14 mm cartridge, Thermo Scientific, Waltham, MA, USA). The mobile phase, consisting of 90% H_3_PO_4_ (23 mM) and 10% methanol, was pumped at a flow rate of 1 mL/min. The injection volume was 40 mL. The UV detector was set at a wavelength of 260 nm. Nicotinamide retention time was about 3 min.

### 3.5. Data Analysis

Data were expressed as the cumulative drug permeation per square centimeter of skin surface. Owing to the sampling of large volumes from the receiver solution and their replacement with equal volumes, the solution was continuously diluted. Taking this into account, cumulative drug permeation (*Q_t_*) was calculated from the following equation:

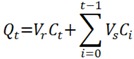

where *C_t_* is the drug concentration of the receiver solution at each sampling time, *C_i_* is the drug concentration of the *i*-th sample, and *V_r_* and *V_s_* are the receiver solution and the sample volumes, respectively.

### 3.6. *In-Vitro* Skin Irritation Test

The *in vitro* skin irritation test was performed at Harlan Biotech Israel according to OECD (Organization for Economic Co-operation and Development) guidelines for the testing of chemicals, test No. 439, adopted July 2010. Microcapsule gel (1 mg/cm^2^ skin) was applied on reconstructed human epidermis, which closely mimics the biochemical and physiological properties of the human skin. Cell viability was measured by enzymatic conversion of the vital dye MTT (3-(4,5-dimethylthiazol-2-yl)-2,5-diphenyltetrazolium bromide) into a blue formazan salt. The observed reduction in the viability of tissues exposed to the microcapsules was compared to negative (Dulbecco’s phosphate-buffered saline) and positive (5% sodium dodecyl sulfate) controls.

### 3.7. Statistical Analysis

Results are presented as the means and standard deviations of at least six determinations. Statistical analysis was performed using an unpaired *t*-test. *p* < 0.05 was considered to be significant.

## 4. Conclusions

The results of this study show that isolated nematocysts of *N. vectensis* can actively deliver hydrophilic drugs such as nicotinamide through the stratum corneum and that accumulation of the drug under the skin can be controlled by microcapsule dosage and the drug exposure time. We have also demonstrated that activated nematocysts can deliver continuously hydrophilic drug as lidocaine HCL. Thus, the microcapsule system can be used as a penetration enhancer independently of the drug applied. An initial safety evaluation indicated that the application is non-irritant. Th**i**s, together with the observed compatibility with hydrophilic drugs, ease of application by topical formulation without an external device, and the submicron diameter of the microcapsules, makes it a promising candidate as a noninvasive application system for continuous drug delivery.
